# Integrating soft robotics and computational models to study left atrial hemodynamics and device testing in sinus rhythm and atrial fibrillation

**DOI:** 10.21203/rs.3.rs-6283242/v1

**Published:** 2025-05-08

**Authors:** Ellen Roche, Manisha Singh, Keegan Mendez, Brian Ayers, Sophie Wang, Atsushi Takahashi, Debbie Teodorescu, Jordi Mill, Carlos Albors, Andreas Escher, Yiling Fan, Caglar Ozturk, Eve Sheridan, Emma Rutherford, Oscar Camara, Tarun Chakravarty

**Affiliations:** Massachusetts Institute of Technology; Institute for Medical Engineering and Science, Massachusetts Institute of Technology; Massachusetts Institute of Technology; Massachusetts General Hospital; Beth Israel Deaconess Medical Center; Massachusetts Institute of Technology; Cedars-Sinai Medical Center; Universitat Pompeu Fabra; Universitat Pompeu Fabra; Massachusetts Institute of Technology; Massachusetts Institute of Technology; Massachusetts Institute of Technology; Massachusetts Institute of Technology; Massachusetts Institute of Technology; Physense, Department of Information and Communication Technologies, Universitat Pompeu Fabra, Barcelona; Cedars-Sinai Medical Center

## Abstract

Atrial fibrillation (AF) poses significant clinical challenges due to the complex and variable geometry of the left atrial appendage (LAA), whose structure complicates the development of personalized interventions like LAA occlusion (LAAO) for stroke prevention Current reliance on animal models and cadavers for the assessment of left atrium (LA) and LAA to study AF-related disease and interventions raises reproducibility concerns, necessitating the development of high fidelity, physiologically relevant tools. To address this, we present a multimodal framework combining a soft robotic benchtop simulator, a lumped parameter model (LPM), and finite element analysis (FEA) to replicate LA function in sinus rhythm, atrial flutter, and AF. The system integrates 3D-printed, patient-specific LA geometries with soft robotic actuators to reproduce realistic wall motion and hemodynamics. A compact, magnetic resonance imaging (MRI)-compatible flow loop, driven by a soft robotic left ventricle (LV), eliminates bulky and non-physiological pulsatile pumps, allowing precise flow measurements and LAAO device testing under clinically relevant conditions. Complementary LPM and FEA models provide mechanistic insights, quantifying systemic hemodynamic changes and LA wall stress during disease and interventions. The models effectively replicate the clinical markers of atrial dysfunction and arrhythmia disorders, and their physiological accuracy is demonstrated through validation against human imaging and porcine models. The soft robotic LV’s ability to drive the mock flow loop is validated in a hybrid synthetic-biological configuration within a swine circulatory system, where the soft robotic ventricle replaces native ventricular contraction to sustain systemic circulation. This scalable and versatile framework integrates experimental and computational techniques to advance cardiovascular biomechanics, supporting device development, clinical research/training, and personalized AF treatment to improve patient outcomes.

## Introduction

Atrial fibrillation (AF) is the most prevalent sustained cardiac arrhythmia, affecting an estimated 33 million people globally and contributing to nearly 20% of ischemic strokes ^1–3^. Characterized by dyssynchronous left atrial (LA) contractions, AF impairs blood flow and predisposes the left atrial appendage (LAA) to thrombus formation ^4^. Left atrial appendage occlusion (LAAO) is a key strategy for stroke prevention in patients with anticoagulant contraindications, but its effectiveness is challenged by the variability of LAA anatomy and the complex hemodynamic changes induced by AF ^5–7^. These challenges highlight the need for physiologically relevant models to study the interplay between LAA anatomy, AF-associated blood flow pattern changes, and intervention outcomes.

Current approaches to testing and evaluating left atrial cardiovascular interventions, including LAAO devices, rely heavily on animal models and cadaveric specimens ^8,9^. While useful, these methods have inherent limitations. Animal models often fail to fully replicate human physiology, raising questions about their translational relevance, while cadaveric models lack the dynamic behavior of living tissues. These shortcomings can delay device testing and regulatory approval processes, increasing costs and extending development timelines. Advanced in vitro and computational models are promising alternatives and are estimated to reduce preclinical costs while accelerating time-to-market ^10–12^. By generating reproducible, (patho)physiologically relevant data, these models offer to address critical clinical research questions and regulatory requirements more effectively ^12^. Thus, there is a need for reproducible, patient-specific platforms that advance cardiovascular biomechanics and hemodynamics research while supporting device design and clinical development in a controlled, physiologically accurate environment. These platforms can also enable personalized treatment planning, procedural optimization, and a deeper understanding of disease mechanisms, addressing critical gaps in cardiovascular research.

Existing cardiac simulators predominantly focus on ventricular function, neglecting the unique biomechanics and hemodynamics of the LA. Atrial contractility and the atrial kick, crucial for ventricular filling and systemic circulation, are absent in most passive models ^13–15^. While recent work has aimed to address some of the limitations by reproducing LA anatomy and hemodynamics ^16^, the absence of active contractility and the atrial kick remains a significant challenge.

To address these gaps, we have developed a multimodal framework that integrates experimental and computational approaches to replicate LA function with high fidelity in both sinus rhythm and AF. Central to this framework is a high-fidelity model of the LA, 3D printed with a soft material and reanimated using soft robotic techniques to replicate patient-specific wall motion and hemodynamics. The system is coupled with a soft robotic left ventricle (LV), which eliminates the need for bulky pulsatile pumps conventionally used in flow loops. Unlike traditional pulsatile pumps, which cause non physiologic expansion of the passive ventricle during systole and contraction during diastole, the soft robotic LV faithfully replicates physiological contraction during systole and relaxation during diastole, ensuring accurate systemic flow dynamics.

To validate the functionality of this model, we used our synthetic, dynamic left ventricular model to replace a native pig ventricle in vivo. Native left ventricular pressure, flow, and systemic blood pressure were initially measured and subsequently reproduced post-euthanasia using the robotic left ventricle integrated into the swine’s circulatory system. The robotic LV was programmed to have the same heart rate as the pre-euthanasia rate and recreated physiologic hemodynamics seen in the native LV, confirming the model’s capability to reproduce physiological hemodynamic conditions. Moreover, the MRI-compatible design of the synthetic model allowed direct imaging and validation of intra-atrial flow dynamics under clinically relevant conditions, demonstrating the resolution of flow recreation achieved by the soft robotic-enabled LA contractile motion.

Additionally, this experimental framework is complemented by computational models, including lumped parameter modeling (LPM) and finite element analysis (FEA). LPM predicts systems-level insights into systemic hemodynamic changes pre- and post-intervention, while FEA provides detailed biomechanical stress distributions and discerns the structural implications of pathologies. Together, these tools quantify key parameters such as LA wall stress and pressure-volume relationship, offering critical insights into hemodynamic adaptations under both physiological and pathological conditions, as well as during therapeutic interventions.

To ensure high fidelity, all three components of the multimodal framework are validated using a combination of human imaging data and porcine animal models. The suite of benchtop and computational models successfully replicated the in vivo structure-function parameters of flow, volume, and pressure for the LA in reservoir, conduit, and pump modes. The benchtop, lumped parameter, and finite element models are presented independently but can potentially be integrated into a hybrid 3D-0D closed-loop framework, enabling simulations in the phantom geometry for direct comparison, cross-validation, and boundary condition exchange.

By fine-tuning circuit and control system parameters and selectively activating individual soft robotic actuators, we simulated diverse atrial reservoir and pump dysfunctions—encompassing contractility, compliance, LAA emptying fraction, atrial flutter, and LAA velocity—and captured their effects on systemic hemodynamics, such as ventricular filling and cardiac outflow. Additionally, we modeled the transcatheter occlusion of the LAA and assessed post-occlusion hemodynamics, highlighting the simulator’s utility for device testing, technology development, and procedural planning. With the growing number of LAAO device options and a certain percentage of technically challenging implants, this framework provides a means to tailor interventions. By enabling patient-specific modeling, it allows for preprocedural strategy optimization, helping clinicians anticipate and address anatomical and hemodynamic challenges before deployment. These models can expedite preclinical trials for LAAO device testing and regulatory approval and reduce reliance on animal testing. As the first soft robotic model of the LA and LAA, it has the potential to create a streamlined pathway for research and development testing toward safe and effective LAAO treatment in AF management.

## Results

### Design of a synthetic, patient-specific soft robotic left atrium and atrial appendage

**Figure 1:**
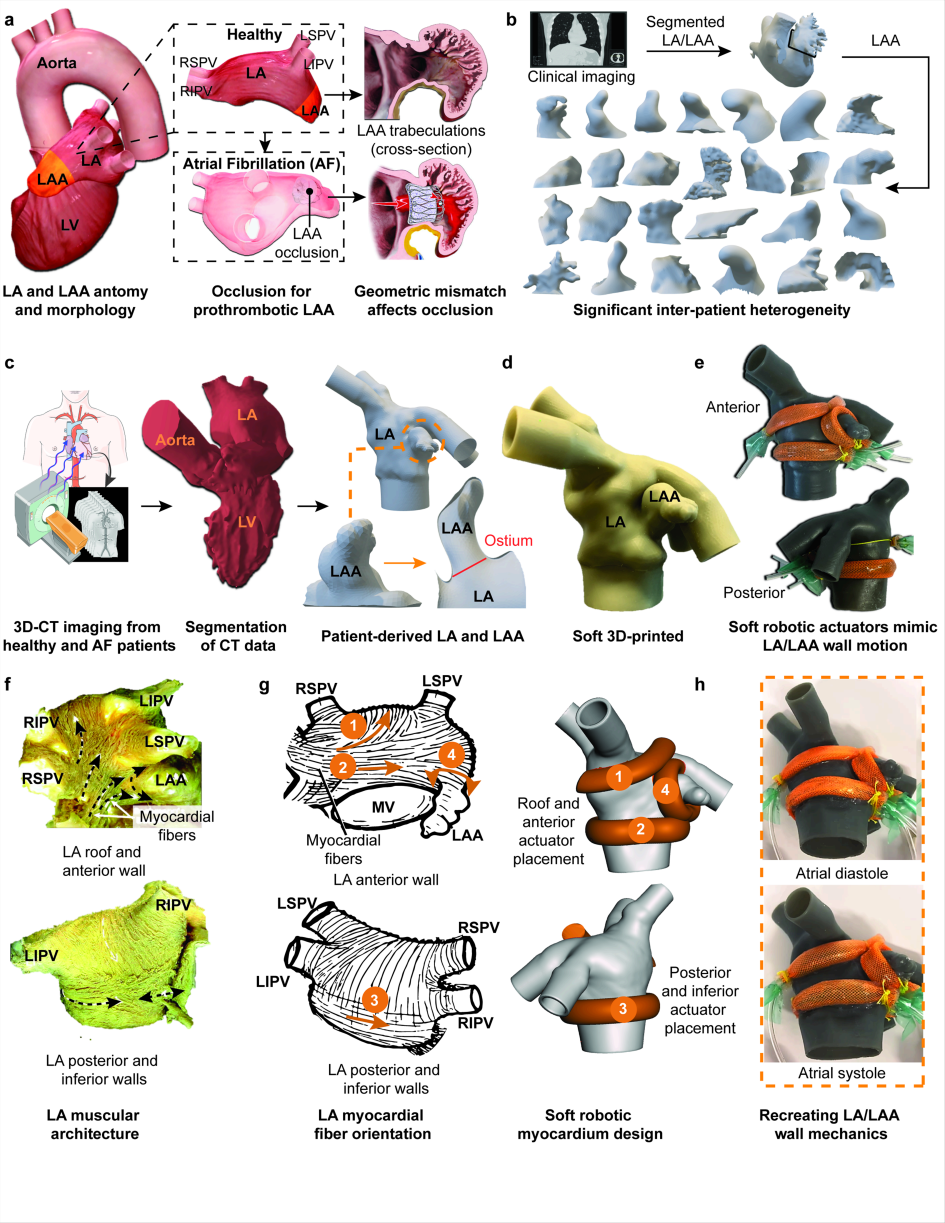
A soft robotic beating heart simulator replicates left atrium (LA) and left atrial appendage (LAA) biomechanics. (a) Anatomical and morphological overview of the LA and LAA, showing healthy and atrial fibrillation (AF) states, along with the challenges posed by geometric mismatches for LAAO due to inter-patient variability and LAA trabeculations. (b) Clinical imaging-derived segmentation of LA and LAA structures from CT data, illustrating significant anatomical heterogeneity across patients. (c) Workflow for 3D-CT imaging and segmentation in healthy and AF patients, producing patient-specific LA and LAA geometries for the simulator design. (d) Soft 3D-printed model of the LA and LAA based on patient data (e) Integration of soft robotic actuators within the LA and LAA model (f) Images showing the muscular architecture of the LA, highlighting the myocardial fiber arrangement, modified from reference 19. (g) Illustrations of native myocardial fiber orientation in the LA and the corresponding placement of soft robotic actuators in four discrete regions, modified from reference 18. (h) Dynamic images of the soft robotic LA and LAA during atrial diastole and atrial systole where inflation and deflation of the soft robotic actuators replicate native wall mechanics.

Atrial fibrillation (AF) is characterized by an irregularly irregular heart rhythm due to uncoordinated atrial signals (Supplementary **Movie S1**). This disorganized activity impairs effective atrial contraction, leading to blood stasis, particularly in the left atrial appendage (LAA), where 90% of thrombi originate ^17^. As a result, LAA occlusion is often necessary for stroke prevention (Fig. 1a). Analysis of patient imaging data confirmed the significant anatomical inter-patient variability of the LAA (Fig. 1b), which contributes to challenges in implantation and peri-device leakage. Patient-specific left atrium models were derived and segmented from high-resolution, time-resolved dynamic computed tomography (CT) scans, allowing for an accurate representation of both healthy and atrial fibrillation-affected geometries, all at the 0% of the R-R interval, corresponding to the onset of ventricular systole. (Fig. 1c). These segmented models were then used to create 3D-printed LA structures using a soft, elastomeric commercial resin including detailed left atrial appendage morphology (Fig. 1d). The resulting models served as the anatomical foundation for the integration of McKibben soft robotic actuators in alignment with the underlying anatomical features (Fig. 1e). These actuators were placed biomimetically, guided by the native myocardial fiber orientations derived from anatomical studies in clinical literature (Fig. 1f). Native myocardial fibers in the LA are organized into distinct bundles with anisotropic alignment, transitioning from oblique to transverse and horizontal in the lower anterior and posterior-inferior walls, and oblique or longitudinal in the roof-anterior wall ^18,19^. These complex orientations facilitate coordinated inward contraction for the atrial kick during systole and stretch-recoil dynamics during diastole, supporting reservoir and conduit functions of the LA for pulmonary venous return. The LAA has an encircling spiral myocardial fiber architecture that generates a wringing motion for effective blood ejection ^18,19^. To mimic these mechanics, actuators were biomimetically placed along the anterior, posterior-inferior, and LAA regions of the LA (Fig. 1f), enabling coordinated contraction, expansion, and wringing motions for realistic atrial and appendage dynamics.

The global myocardial fiber orientation map was simplified into four discrete regions of interest to optimize actuator placement and provide precise control over contractile motion (Fig. 1g). Digital photos of the assembled soft robotic LA model demonstrate the anatomical accuracy and the integration of the soft robotic actuators, showing their placement along the atrial and appendage walls (Fig. 1h). The actuators enabled biomimetic wall motion, including atrial contraction (“kick”) during the systolic phase and relaxation during diastole, simulating native atrial pumping and filling functions.

**Figure 2:**
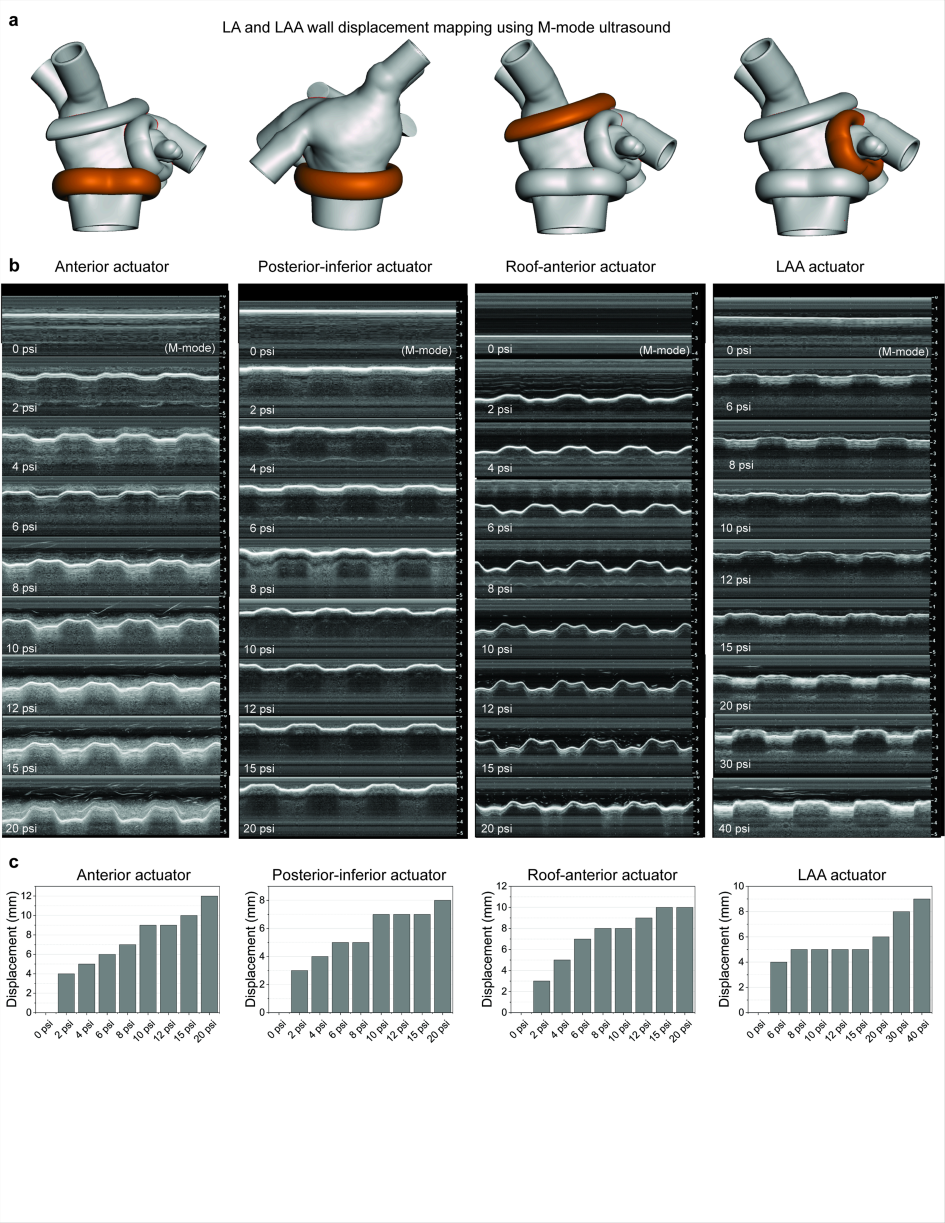
Soft robotic actuator positioning influences left atrium (LA) and left atrial appendage (LAA) wall motion and displacement under varying pressures. (a) Different configurations of soft robotic actuators wrapped around the LA and LAA structures at distinct anatomical orientations (highlighted in orange), impacting the extent of wall motion and direction of simulated atrial contraction. (b) M-mode ultrasound images capturing wall displacement at increasing actuator pressures. (c) Quantitative analysis of wall displacement in response to input pressure to each actuator, highlighting tunable mechanical response.

The motion generated from the placement of actuators along these key regions, guided by fiber orientation, was validated through M-mode ultrasound imaging (Fig. 2a). The input pressure for the actuators was systematically varied from 0 to 40 psi to assess the range of wall displacement. Ultrasound imaging (Fig. 2b) confirmed the effectiveness of individual actuators in producing tunable wall motion. For the LA wall, actuators achieved displacements ranging from 2 to 12 mm with input pressures of 2 to 20 psi and for the LAA wall, displacements of up to 9 mm were achieved with pressures up to 40 psi (Fig. 2c). These results demonstrated that the actuators could reliably replicate both healthy and impaired contractile behaviors, providing the flexibility to model a variety of atrial pathophysiologies. This biomimetic contractile motion can support the ejection of blood from the LA and LAA and prevent stagnation, critical for reducing thrombus formation in healthy physiology. We can also mimic diseased conditions such as AF, where the LA and LAA lose the coordinated contractile motion, leading to blood stasis and an increased risk of thromboembolism.

### Recreating healthy left-sided hemodynamics with atrial contraction

The soft robotic left atrium model was integrated with a soft robotic left ventricle model, both segmented from dynamic CT imaging data, to create a comprehensive system capable of reproducing left-sided cardiovascular hemodynamics (Supplementary **Movie S2**). The design and placement of actuators in the LV model were previously optimized through computational and experimental methods, ensuring realistic contractile motion and hemodynamic performance ^20,21^. The soft robotic LV eliminates the need for an external pulsatile pump by generating systemic cardiovascular hemodynamics on its own. While the primary focus of this work is on recreating pressures and flows associated with the LA, including the precise replication of the “atrial kick” and A-wave characteristic of atrial contraction, the use of a patient-specific dynamic LV model reinforces the system’s ability to replicate the entire left-sided cardiac and circulatory hemodynamics. This integration is critical for the successful development of an LA simulator, as it allows for studying the effects of atrial contraction and wall mechanics on overall cardiac function in both healthy and diseased states.

**Figure 3:**
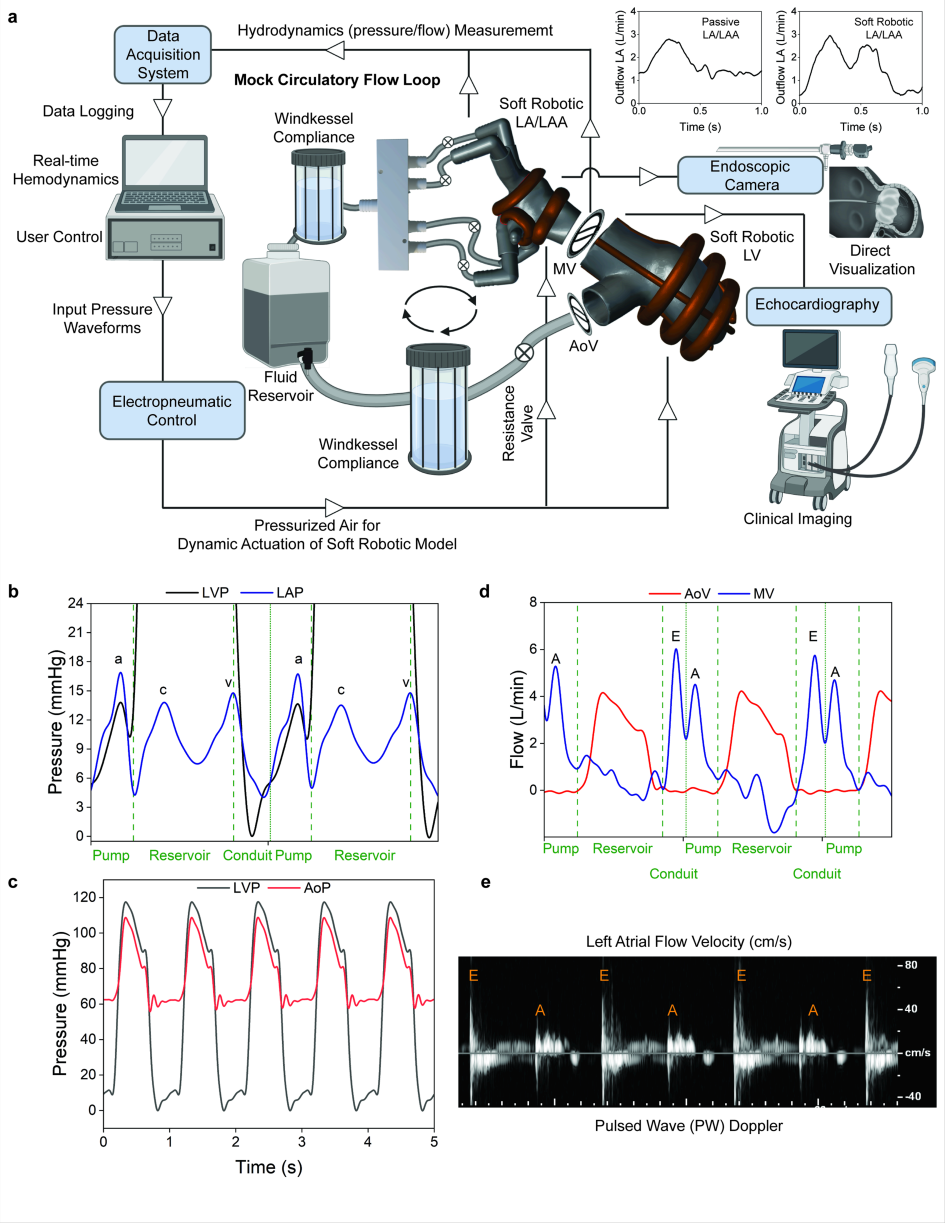
A mock circulatory flow loop enables hemodynamic measurements in the soft robotic left heart simulator. (a) Schematic of the mock circulatory flow loop with components to replicate cardiac and vascular hemodynamics, including the soft robotic left atrium (LA) left atrial appendage (LAA), and left ventricle (LV) models. The circuit includes mechanical mitral valve (MV) and aortic valve (AoV) components, which are essential for simulating unidirectional flow. (b) Pressure waveforms from the left ventricular pressure (LVP) and left atrial pressure (LAP) within the circuit, showing characteristic phases of the cardiac cycle (a, c, v waves) associated with left atrial hemodynamics. (c) Pressure traces show LVP and aortic pressure (AoP) over several cardiac cycles, highlighting the model’s capacity to simulate systemic hemodynamics. (d) Flow waveforms through the aortic (AoV) and mitral (MV) valves. (e) Pulsed-wave Doppler of LA flow velocities capturing the hemodynamic characteristics of the LA to mimic LA filling and emptying patterns.

The mock circulatory flow loop was specifically designed to simulate systemic circulation, incorporating adjustable parameters such as preload, afterload, vascular compliance, and resistance (Fig. 3a, supplementary **Figure S1**). The system utilized two clinically standard mechanical valves (mitral and aortic) to ensure unidirectional flow through the circuit, with the soft robotic LV functioning as the primary pump to drive fluid flow (supplementary **Figure S2**). The LA and LV models were controlled using a custom electropneumatic control box (supplementary **Figure S3**), enabling synchronization of atrial and ventricular contraction at clinically relevant heart rates (e.g., 40–150 bpm). The loop featured integrated pressure and flow sensors for real-time hemodynamic measurements and the model was equipped with a port for an endoscopic camera, providing visualization of endocardial structures. The actuation of soft robotic elements on the LA and LAA successfully replicates the atrial kick, a dynamic contraction crucial for active ventricular filling, which cannot be achieved with passive 3D-printed models (supplementary **Figure S4**). Figure 3b and supplementary **Figure S5** highlight the physiologic left atrial pressure waveforms generated by the system, including the characteristic a-, c-, and v-waves, corresponding to the pump, reservoir, and conduit phases of the LA cycle. These pressure waveforms were synchronized with the LV contraction to replicate realistic cardiac cycles, demonstrating their ability to simulate the functional interplay between atrial contraction and ventricular filling. The a-wave reflects atrial contraction during late ventricular diastole, the c-wave represents a transient increase in left atrial pressure caused by ventricular contraction and mitral valve bulging, and the v-wave arises from passive atrial filling during ventricular systole, collectively illustrating the pump, reservoir, and conduit phases of the left atrial cycle ^22^. The soft robotic LV generated biphasic ventricular pressures (120/6 mmHg) and drove systemic circulation, producing phasic aortic systemic pressures of 115/60 mmHg at a sinus rhythm of 60 bpm (Fig. 3c). When combined with the soft robotic LA, the system reproduced physiologically accurate flow waveforms for left-sided circulation. Figure 3d demonstrates alternating mitral and aortic flow, with a cardiac outflow of over 5 L/min. The mitral valve flow waveform exhibited distinct E and A wave regions, representing passive ventricular filling during early diastole and active filling driven by atrial contraction, respectively. These findings confirm the capability of the soft robotic LA to contribute to active ventricular filling, mimicking the functional role of atrial contraction in the cardiac cycle. The soft robotic LA-LV system is compatible with clinical imaging modalities such as echocardiography (Fig. 3e). Pulsed wave Doppler imaging was used to measure fluid flow velocities in the soft robotic LA, visualizing E and A wave patterns associated with mitral flow ^23^. This capability allows for real-time, clinically relevant assessments, making the system suitable for studying both physiological and pathological atrial mechanics and hemodynamics in a controlled environment.

### Multimodality imaging confirms mimicry of physiological wall and valve function

**Figure 4:**
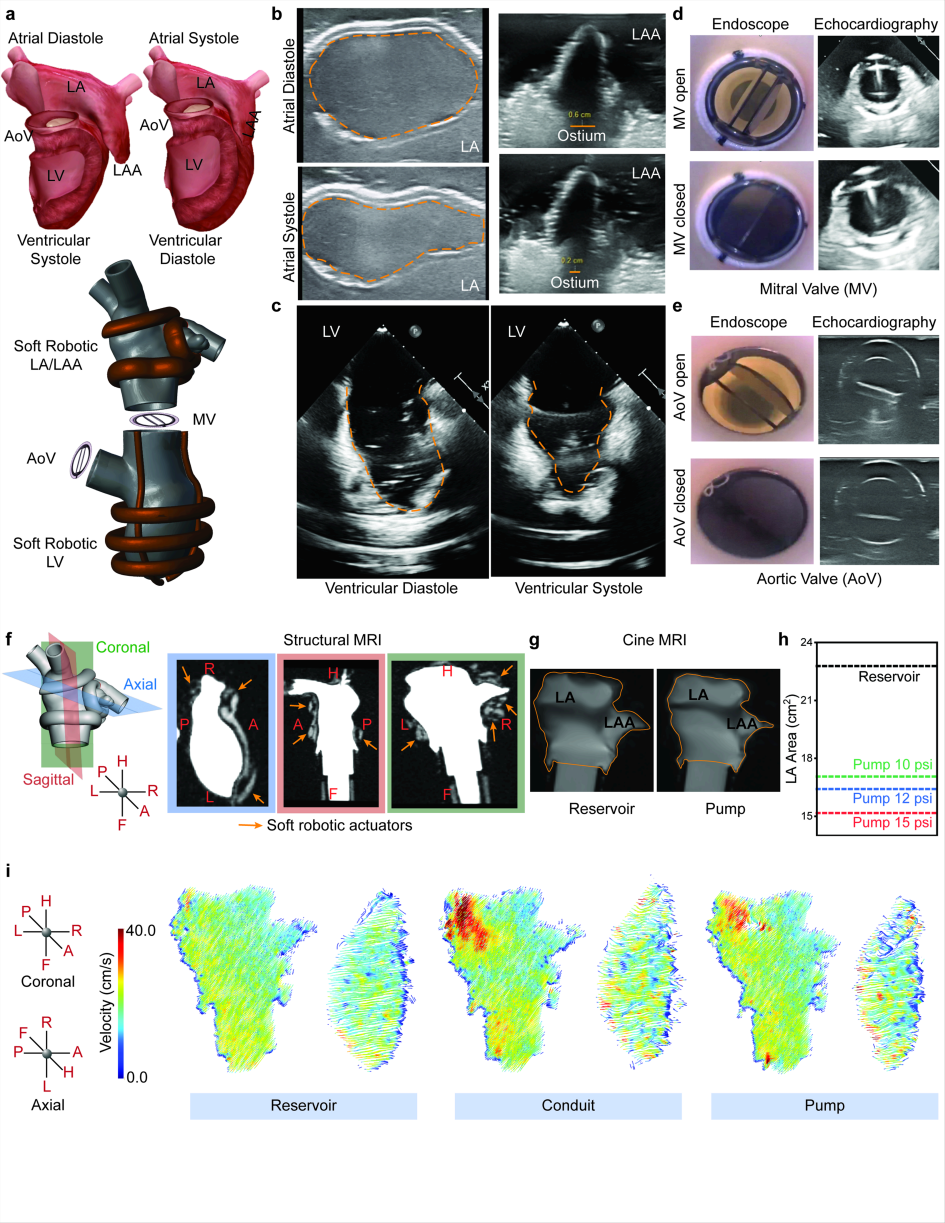
Functional assessment of the soft robotic left atrium (LA), left atrial appendage (LAA), and left ventricle (LV) model demonstrates its ability to replicate realistic cardiac hemodynamics and valve mechanics. (a) Soft robotic LA/LAA and LV models equipped with actuators designed to simulate contraction and relaxation with high anatomical and physiological fidelity shown in top panels. (b) Ultrasound images of the LA and LAA during atrial diastole and systole to replicate physiological changes in atrial geometry and chamber volumes. (c) Echocardiographic images capturing the soft robotic LV during ventricular diastole and systole, showing motion patterns that replicate physiological LV contraction and relaxation. (d) Visualization of the mitral valve (MV) using endoscopic and echocardiographic imaging in both open and closed states, demonstrating functional valve dynamics. (e) Endoscopic and echocardiographic views of the aortic valve (AoV) in open and closed states, driven by soft robotic LV. (f) Magnetic resonance imaging (MRI) orientation setup and structural MRI images showing coronal, sagittal, and axial views of the LA and LAA in the soft robotic model. The locations of the soft robotic actuators are indicated by orange arrows. (g) Cine MRI images capturing LA and LAA structure under reservoir and pump conditions. (h) Area measurements of the LA under different pressures (10, 12, and 15 psi) during atrial contraction. The reservoir state (non-actuated) is used as a reference for the maximal LA area. (i) Velocity-encoded 2D MRI images showing blood flow velocity vectors in the LA and LAA under reservoir, conduit, and pump conditions.

The biomimetic, 3D-printed design of the soft robotic models successfully replicates the anatomy and physiological motion of the left atrial and ventricular wall and the function of heart valves (Fig. 4a, Supplementary **Movie S3**). Epicardial echocardiography was employed to capture real-time images of the LA and LAA during their dynamic actuation in tandem with the soft robotic LV and mechanical valves. As shown in Fig. 4b, the echocardiographic images illustrate the displacement of the LA wall between atrial diastole and systole. During atrial systole, the inflation of the soft robotic actuators mimics native atrial contraction, resulting in inward displacement of the LA wall. Conversely, deflation during diastole allows the wall to return to its resting position, replicating the passive reservoir phase of the LA. This biomechanical and functional mimicry, enabled by the inflation and deflation of the soft robotic actuators, aligns closely with physiological motion patterns observed in the native atrium. Furthermore, the LAA ostium shows a measurable reduction (> 66%) in diameter during systole, as highlighted in the echocardiographic images, effectively replicating the contractile motion that promotes blood ejection from the LAA. Figure 4c highlights the synchronized function of the soft robotic LV and LA. Echocardiographic images captured during ventricular systole and diastole illustrate the ability of soft robotic muscles to create coordinated motion of the LV wall, mirroring native ventricular contraction and relaxation. This synchronization, achieved through the electropneumatic control system, enables a seamless interplay between the LA and LV, ensuring effective blood flow within the system. Endoscopic and echocardiographic images confirm the opening and closing of the valves in synchrony with the actuation of the soft robotic LA and LV. During ventricular diastole, the mitral valve (MV) opens to facilitate passive filling of the LV, as seen in the clear circular aperture in Fig. 4d. During systole, the MV closes tightly to prevent retrograde flow, ensuring unidirectional blood movement. Similarly, the aortic valve (AoV) exhibits characteristic behavior, opening fully during ventricular systole to allow forward blood flow into the systemic circulation and closing during diastole to maintain systemic pressure (Fig. 4e). Figure 4f shows the anatomical orientation of the soft robotic LA/LAA model in coronal, sagittal, and axial planes, with structural MRI highlighting the placement of the actuators (orange arrows). Cine MRI images (Fig. 4g) further illustrate the dynamic wall motion between reservoir and pump phases, with the LA area changing significantly during systole and diastole, as quantified in Fig. 4h. Wall displacement driven by input actuation pressures of 10, 12, and 15 psi resulted in LA long - axis cross-sectional area variations between 15 and 23 cm^2^, replicating physiologically relevant atrial reservoir and pump phases ^24^. These results demonstrate that the soft robotic myocardial substitute can faithfully replicate the physiological wall motion of the LA and LAA, as well as the dynamic functionality of the mitral and aortic valves. The system’s compatibility with clinical imaging modalities, such as MRI, echocardiography, and endoscopy, further highlights its potential as a platform for studying cardiac wall and valve mechanics in both healthy and pathological states.

### Intra-atrial flow velocity measurement via phase-contrast MRI

**Figure 5:**
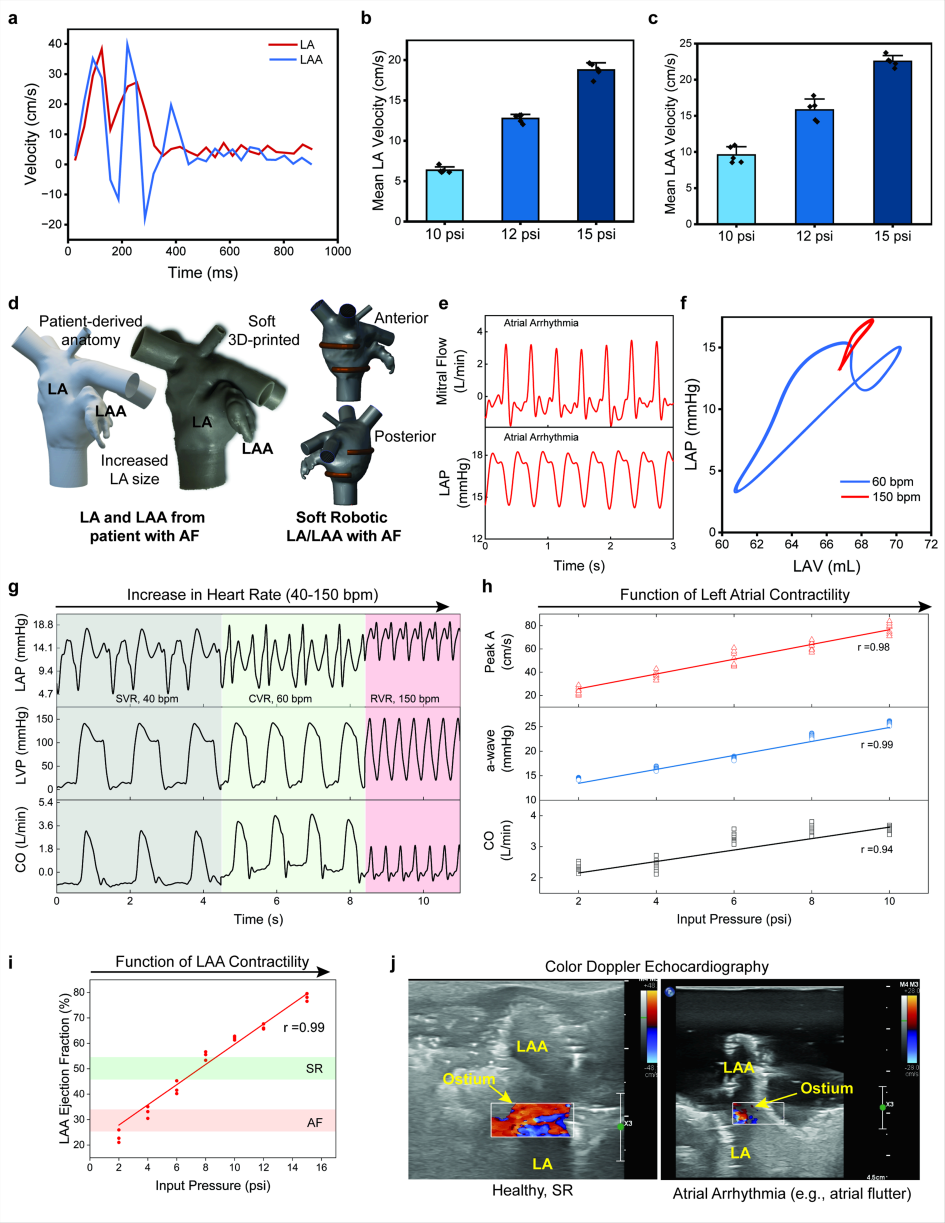
Simulation of atrial fibrillation (AF) in the soft robotic left atrium (LA) and left atrial appendage (LAA) model demonstrates hemodynamic changes, contractility, and flow patterns associated with AF and sinus rhythm (SR). (a) Temporal velocity profile of LA and LAA flow velocities over time in the soft robotic model measured via 2D phase-contrast magnetic resonance imaging (MRI). (b) Mean LA velocity under increasing actuation pressures (10, 12, and 15 psi). (c) Mean LAA velocity under the same range of pressures (10, 12, and 15 psi). (d) Development of the soft robotic LA and LAA model derived from a patient with AF, showing enlarged LA and LAA geometries characteristic of AF-induced remodeling. (e) Mitral flow and left atrial pressure (LAP) waveforms associated with atrial arrhythmia. (f) Pressure-volume (PV) loops of the LA in sinus rhythm (60 bpm) vs. atrial flutter (150 bpm). (g) Hemodynamic response to increasing heart rates (40–150 bpm), showing LAP, left ventricular pressure (LVP), and cardiac outflow (CO), associated with atrial arrhythmias, including slow ventricular response (SVR, 40 bpm), controlled ventricular response (CVR, 60 bpm), and rapid ventricular response (RVR, 150 bpm). (h) Graphs depicting the effect of LA contractility as a function of input pressure on peak A-wave velocity, a-wave pressure, and cardiac outflow (CO). (i) LAA ejection fraction as a function of input pressure (or LA/LAA contractility), mimicking states of both AF and SR. (j) Color Doppler echocardiography images showing simulation of flow through the LAA ostium in healthy SR vs. atrial flutter.

Conventional rigid benchtop simulators lack the dynamic wall motion required to replicate physiological intra-atrial flow energetics, limiting their ability to provide meaningful insights. In contrast, the soft robotic LA/LAA model, with its MRI compatibility and high anatomical and functional precision, allows for the visualization and quantification of flow patterns and velocities within the LA and LAA under sinus rhythm using a 3T MRI system (supplementary **Figure S6**). Phase-contrast 2D flow imaging sequences were utilized to measure intra-atrial velocities and analyze flow patterns generated by the soft robotic actuators (Fig. 4i). The flow streamlines show that fluid flow velocity was the highest (35–40 cm/s) at the right pulmonary vein junction and the blood flow was also slower (10–15 cm/s) at the left pulmonary vein junction, a pattern also seen in human patients in literature ^25^. During the reservoir phase, peak flow velocities were lower (15–25 cm/s) and uniformly distributed, while the conduit and pump phases exhibited higher peak velocities (30–40 cm/s) driven by LV suction and atrial contraction (atrial kick), mirroring physiological behavior ^26^. Velocity-time graphs (Fig. 5a) show dynamic velocity changes within the LA and LAA throughout the cardiac cycle, with peak velocities in the range of 30–40 cm/s, consistent with literature ^27^. Mean velocity measurements across actuation pressures (10, 12, and 15 psi) for the LA (Fig. 5b) and LAA (Fig. 5c) further confirmed the system’s ability to generate physiologically relevant flow velocities. At 15 psi, the mean velocities reached approximately 18 cm/s in the LA and 24 cm/s in the LAA, consistent with reported values in atrial (patho)physiology ^26,28,29^. This study demonstrates that the integration of soft robotics into the model enables the accurate reproduction of clinically relevant intra-atrial and appendage flow velocities, which are critical for replicating flow behaviors, including stagnation, observed in pathological conditions such as atrial fibrillation.

### Replicating pathological hemodynamics of atrial arrhythmias

This study demonstrates the ability of the soft robotic LA/LAA model to replicate pathophysiological hemodynamics characteristic of atrial arrhythmias, such as atrial flutter or fibrillation, including reduced atrial contractility and its associated impact on ventricular filling and cardiac outflow (CO). By modulating the actuation input pressure on the soft robotic actuators and varying actuation rates, the model effectively mimics the lack or reduction of atrial contractility observed during atrial arrhythmias and provides insights into the resulting hemodynamic alterations. As shown in Fig. 5d, the patient-specific LA and LAA morphology associated with AF was recreated, featuring an enlarged atrium and diminished myocardial contractility, mimicked by incorporating only two soft robotic actuators. Figure 5e demonstrates the replication of mitral flow velocities and left atrial pressures during atrial arrhythmias (e.g., atrial flutter), with a noticeable absence of strong atrial contraction (atrial kick). The pressure-volume loop (Fig. 5f) further mimics the impact of atrial arrhythmias on LA hemodynamics. Under sinus rhythm, the PV loop exhibits characteristic figure-eight a- and v-loops, corresponding to active atrial contraction (a-wave) and passive atrial filling (v-wave). In contrast, during atrial flutter, the PV loop is reduced to a v-loop alone, indicating the absence of active atrial contribution.

By varying heart rates from 40 to 150 bpm, the model captured the hemodynamic changes associated with slow ventricular response (SVR), controlled ventricular response (CVR), and rapid ventricular response (RVR) during atrial arrhythmias. Figure 5g demonstrates that as heart rate increased, left atrial pressures (LAP), left ventricular pressures (LVP), and CO were significantly altered. During SVR (40 bpm), LAP and CO remained within physiological ranges due to sufficient ventricular filling time, but LVP showed a pronounced relaxation peak, highlighting impaired LV relaxation dynamics. Under CVR (60–80 bpm), normal atrial contraction maintained efficient LAP, LVP, and CO. However, during RVR (150 bpm), the reduced ventricular filling time led to diminished CO, increased diastolic LVP, and abnormal LAP dynamics, closely mimicking the hemodynamic effects of rapid atrial arrhythmias.

We quantified the influence of LA contractility on ventricular filling and cardiac outflow by systematically varying the actuation input pressure of the LA soft robotic actuators. Figure 5h shows that increasing input pressure led to greater mitral flow peak *A* velocities, higher LAP peak *a* values, and enhanced CO, with strong correlations (r > 0.94) between these parameters and LA actuation pressure. This finding highlights the importance of atrial contractility in maintaining efficient ventricular filling and systemic outflow. The effect of LAA contractility on LAA ejection fraction was assessed by varying the LAA actuation input pressure. As shown in Fig. 5i, higher actuation pressures correlated with increased LAA ejection fraction, transitioning from impaired emptying observed in AF to healthy ejection levels characteristic of sinus rhythm (SR). The ability to replicate this pathophysiological feature emphasizes the utility of the model in studying LAA dynamics and potential thrombus formation in conditions such as AF ^30–32^. Color Doppler echocardiography (Fig. 5j) provided a real-time visualization of simulated LAA flow under both healthy and pathological conditions. In healthy SR (60 bpm), ordered flow through the LAA ostium was observed, whereas atrial flutter (150 bpm) demonstrated reduced flow across the LAA ostium, a known contributor to thrombus formation and embolic risk.

### Device sizing and placement for LAA occlusion in patient-specific models

**Figure 6:**
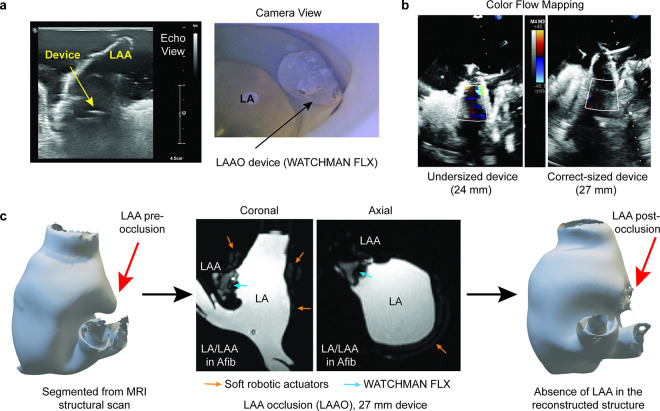
Assessment of left atrial appendage occlusion (LAAO) in the soft robotic simulator demonstrates device positioning and flow dynamics. (a) Imaging and visualization of the LAAO device (WATCHMAN FLX) within the LAA of the soft robotic simulator. Echocardiographic and camera views show the proper positioning of the device within the LAA. (b) Color flow Doppler mapping of the LAA with undersized (24 mm) and correctly sized (27 mm) occlusion devices. (C) Phase contrast magnetic resonance imaging (MRI)-based segmentation and imaging of the LAA and LA before and after LAAO. Orange arrows show the integrated soft robotic actuators around the underlying anatomy and blue arrows highlight the deployed WATCHMAN FLX device for LAAO.

To demonstrate testing and optimizing device sizing and placement for LAAO in patient-specific anatomies using our simulator, occlusion devices were deployed into the LAA to evaluate their performance and assess the degree of closure. For this patient-specific model, two WATCHMAN FLX^™^ devices, sized 24 mm and 27 mm, were tested by an operator guided by echocardiography (Fig. 6a). Real-time echocardiography and endoscopy imaging allowed precise placement of the devices. Supplementary **Movie S3** shows the WATCHMAN device functioning within the pulsatile environment of the soft robotic LA model. The level of occlusion achieved by the devices was assessed using 2D color Doppler imaging (Fig. 6b). The 24 mm device showed incomplete occlusion, evidenced by the presence of color jets behind the device, indicating residual flow through the LAA. In contrast, the 27 mm device demonstrated effective occlusion, with no visible color jets, confirming more complete closure of the LAA. This highlights the importance of proper device sizing in achieving optimal occlusion and validates the ability of the model to assess device performance in real-time.

To further confirm appropriate device placement, a phase contrast MRI was performed, providing detailed visualization of the implanted device within the LAA (Fig. 6c). The gray shading in the MRI images represents the implanted device, clearly visible within the LAA. Post-implantation analysis showed the absence of the LAA in the reconstructed fluid volume, indicating successful closure with the 27 mm device. This study highlights the utility of the patient-specific soft robotic simulator to guide device placement in complex anatomies, providing operators with a platform to practice and optimize deployment strategies in realistic clinical scenarios.

### Lumped parameter modeling of LAA occlusion effects on LA hemodynamics

**Figure 7:**
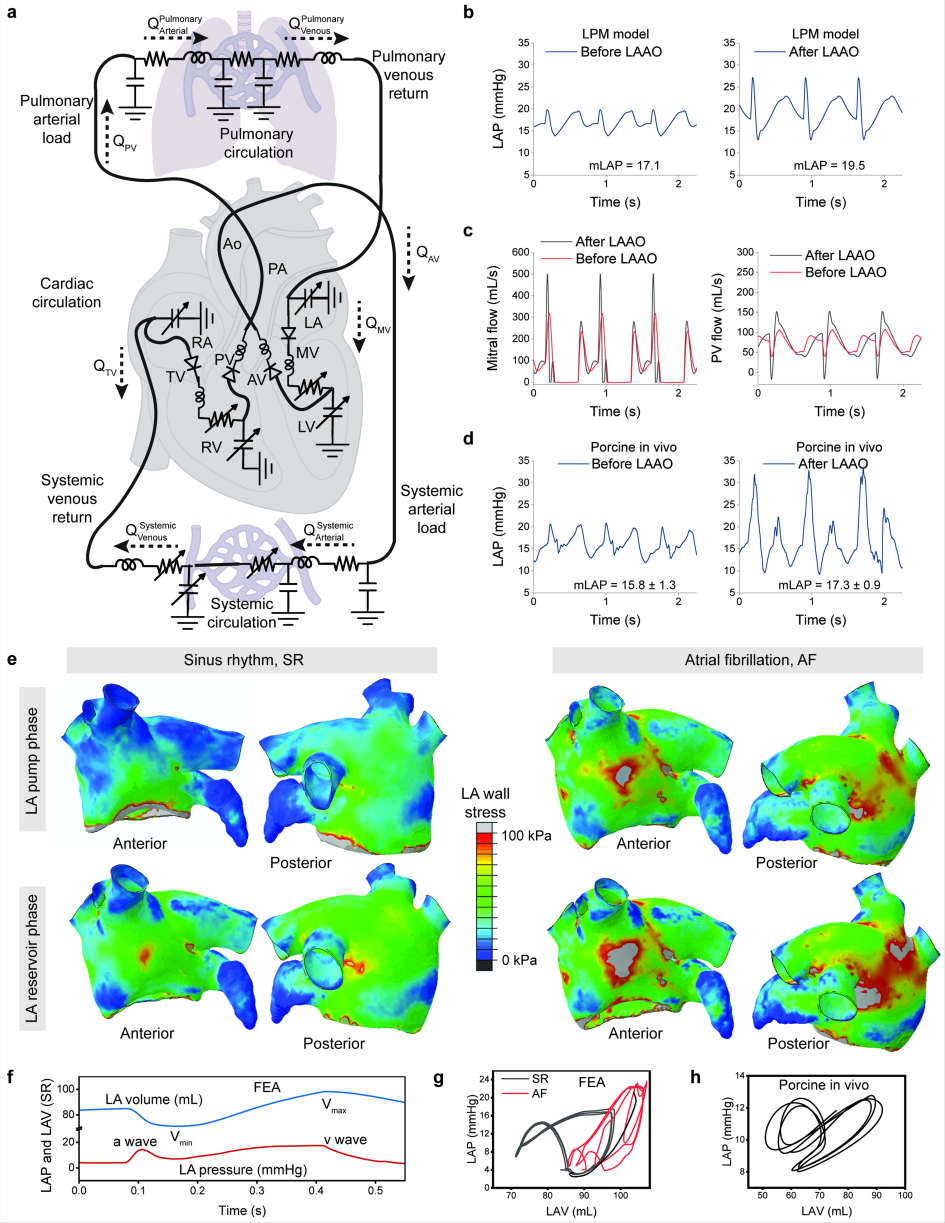
Computational models analyze the impact of left atrial appendage occlusion (LAAO) on left atrial (LA) hemodynamics and the effect of atrial fibrillation (AF) on LA wall mechanics. (a) Schematic of the lumped parameter model (LPM) developed to study and understand the effect of LAAO on LA hemodynamics. (b) Hemodynamic data from the LPM model, showing left atrial pressure (LAP) before and after LAAO. (c) Mitral and pulmonary vein flow before and after LAAO. (d) In vivo LAP data from live porcine models before and after LAAO, showing a similar increase in mLAP post-occlusion, aligning with predictions from the LPM. (e) Predicted LA wall stress distributions during the pump and reservoir phases of the cardiac cycle in sinus rhythm (SR) and AF, derived from a dynamic finite element analysis (FEA) model. The gray color in the contour plots indicates values that exceed the maximum range of the color bar. (f) Simulated LAP and LA volume (LAV) during SR, showing characteristic a- and v-waves with corresponding minimum (Vmin) and maximum (Vmax) LA volumes. (g) Pressure-volume loops for SR and AF generated by FEA model. (h) Validation of simulated pressure-volume loop shape with in vivo data from a healthy porcine heart in SR.

To understand how left atrial appendage occlusion affects left atrial filling and emptying, a lumped parameter model was developed. The model simplifies the cardiovascular system into a set of ordinary differential equations that represent pressure-volume relationships, flow dynamics, and vascular resistances. Figure 7a illustrates the electrical analog of the LPM, which models the systemic and pulmonary circulations as a network of resistances and capacitances, allowing for the simulation of LAAO effects on LA hemodynamics. Figure 7b compares the mean left atrial pressure (mLAP) before and after LAAO, showing that the model predicted an increase in mLAP from 17.1 mmHg to 19.5 mmHg post-LAAO. This reflects the added load on the LA due to reduced reservoir function of the LAA, consistent with clinical observations of mild elevations in LA pressure following occlusion ^33^. The mitral flow waveform revealed a slight increase in peak velocities post-LAAO, potentially suggesting an enhanced atrial contribution to ventricular filling (Fig. 7c). The PV flow waveform, however, displayed a subtle increase in backward flow (negative velocities), indicating slight retrograde flow into the pulmonary veins during atrial contraction post-LAAO.

To validate the LPM, measurements from a porcine in vivo model were compared to the simulated results (Fig. 7d). The in vivo data showed a similar increase in mLAP from 15.8 ± 1.3 mmHg before LAAO to 17.3 ± 0.9 mmHg after LAAO, closely aligning with the LPM predictions. The waveforms for LAP also showed comparable patterns, confirming the model’s accuracy in replicating LA hemodynamics and studying the nuanced effects of LAAO in a closed-loop cardiovascular system. This increase in mLAP has also been reported in clinical literature in human patients^33,34^.

### Finite element analysis of atrial fibrillation impact on LA mechanics

The impact of AF on left atrial wall mechanics and pressure-volume hemodynamics was investigated using a dynamic finite element analysis model based on the SIMULIA Living Heart Project. The model integrates realistic electrical, structural, and fluid flow physics to simulate dynamic response of the heart, enabling the study of coupled electromechanical behavior where electrical excitation drives mechanical contraction. Following the electro-mechanical simulation of sinus rhythm, the model was modified to represent AF (as described in the Methods section) and analyzed over three sequential irregular cardiac cycles.

Predicted LA wall stress during its function as a conduit, pump, and reservoir is presented in Fig. 7e for both SR and AF. Relatively uniform stress distributions were observed during SR, reflecting healthy tissue mechanics and functional coordination. In contrast, AF disrupted these coordinated mechanics, leading to irregular and elevated stress distributions. Results demonstrate a marked increase in LA wall stress during AF, specifically, mean LA wall stress over a cardiac cycle increased from 3.17 kPa in SR to 19.54 kPa in AF. The loss of mechanical coordination during AF created stress concentration zones, which are known to act as sites of microtrauma or remodeling ^35^. These high-stress regions can predispose the atrium to structural abnormalities such as dilation, wall thinning, and fibrotic tissue deposition, potentially contributing to the progression of AF ^35–37^. Stress patterns derived from this model can offer predictive potential, identifying regions at risk for remodeling and the likelihood of progression from paroxysmal to persistent AF. Such insights could be instrumental in planning mechanism-based interventions. For example, identifying high-stress concentration regions could guide catheter ablation strategies to target potential triggers or contributors to AF.

In addition to wall stress, LA pressure and volume data were analyzed to evaluate hemodynamic differences between SR and AF and their correlation with wall stress (Fig. 7f). The analysis revealed that AF significantly increased mean LA pressure, rising from 7.04 mmHg in SR to 14.49 mmHg in AF (Fig. 7g). The pressure-volume curve demonstrated the characteristic figure-eight shape in SR, while the a-loop was absent in AF. Elevated wall stress and stiffness were associated with reduced compliance, leading to a steeper pressure-volume curve in SR compared to AF (Fig. 7g). The shape of the pressure-volume loop generated by the FEA model was further validated by pressure-volume measurements obtained from a healthy live porcine in SR (Fig. 7h). By integrating pressure-volume analysis with wall stress data, the model highlights the interplay between mechanical and hemodynamic dysfunction in AF. This model could be adapted to patient-specific geometries in the future, enhancing its clinical utility. The benchtop, computational lumped parameter, and finite element models are presented as separate analyses; however, future integration into a hybrid experimental-computational framework could enable 3D-0D coupled closed-loop modeling. Simulations could be conducted in the same geometry as the phantom, allowing for direct comparisons, cross-validation, and the use of one model’s outputs as boundary conditions for another.

### Integration and validation of the soft robotic LV in swine circulatory system

**Figure 8:**
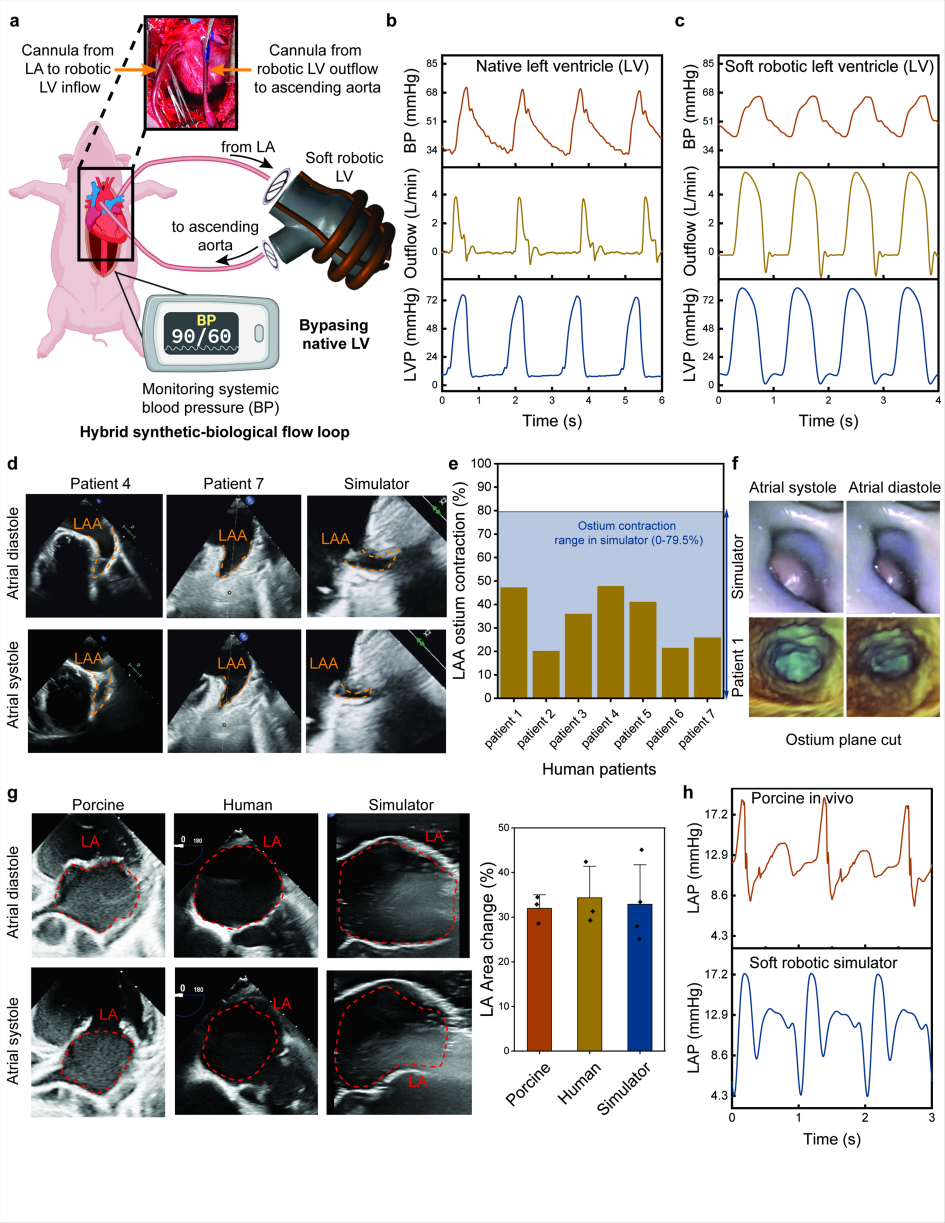
The soft robotic left atrium (LA), left atrial appendage (LAA), and left ventricle (LV) models were validated against porcine and human data, demonstrating realistic hemodynamics and biomechanics. (a) Experimental setup for testing the soft robotic LV’s ability to replicate systemic physiological flow and pressure parameters under physiological resistance, compliance, and native vasculature with a hybrid synthetic-biological flow loop configuration in a swine circulatory system. (b) Invasive hemodynamic data recorded in a live swine with the native LV: systemic blood pressure (BP), cardiac outflow, and left ventricular pressure (LVP). (c) Hemodynamic measurements with the soft robotic LV, showing BP, cardiac outflow, and LVP generated by the synthetic LV inside the porcine circulatory system with native vasculature. (d) Echocardiographic images comparing LAA ostium motion in two human patients (Patients 4 and 7) and the soft robotic simulator during atrial systole and diastole, closely match the dynamics observed in human cases. (e) Quantitative analysis of LAA ostium contraction in human patients compared with the range achieved by the soft robotic simulator. (f) Views of the LAA ostium plane in the simulator (endoscopic camera) and in a human patient (Patient 1, echocardiography) during atrial systole and diastole. (g) Comparison of LA area changes across porcine, human, and LA simulator models via echocardiographic images in atrial diastole and systole show comparable area changes in all models. The bar graph shows quantitative LA area contraction, with the simulator showing close alignment with both porcine and human data, indicating accurate replication of atrial mechanics. (h) Left atrial pressure (LAP) traces in porcine in vivo vs. soft robotic simulator.

To validate the functionality of the soft robotic left ventricle model to drive systemic hemodynamics, it was integrated into a swine circulatory system, forming a hybrid synthetic-biological configuration. As shown in Fig. 8a, the native LV was bypassed, and the soft robotic LV was connected to the native vasculature via inflow cannulas from the LA to the robotic LV and outflow grafts from the robotic LV to the ascending aorta. Systemic blood pressure and invasive left-sided hemodynamics were first recorded in a live porcine model with native LV (Fig. 8b). The animals were then euthanized and the setup aimed to replicate the function of the native left-sided heart by pumping blood systemically with soft robotic LV.

The soft robotic LV actuation was triggered using a custom electro-pneumatic control system at the matching heart rate to the data collected in the living model, ensuring seamless comparison between the biological and synthetic LV. Figure 8c demonstrates the hemodynamic performance of the soft robotic LV, generating clinically relevant systemic blood pressures (≈ 68/40 mmHg), ventricular outflow (≈ 4 L/min), and left ventricular pressures (≈ 75/2 mmHg). The soft robotic LV successfully sustained systemic circulation, replicating the hemodynamic profiles of the native LV, including the phasic patterns of pressure and flow. The successful validation of the soft robotic LV into a swine circulatory system establishes its capability to replicate left-sided cardiac function under physiological conditions and provides a versatile platform for its use in mock flow loops for preclinical studies to model and address complex cardiovascular conditions and test interventions.

### Comparison against human imaging and porcine hemodynamic data

The soft robotic LA and LAA model was validated against clinical imaging data from human patients and hemodynamic data from a porcine model, demonstrating its accuracy in replicating physiological and pathological atrial mechanics and hemodynamics. Figure 8d compares the LAA wall motion in human patients (Patient 4 and Patient 7) with that of the simulator during atrial diastole and systole. The echocardiographic images show a close match in LAA contraction dynamics, with the simulator accurately replicating the inward contraction of the LAA ostium observed in human imaging. Quantification of LAA ostium contraction percentages (Fig. 8e) further confirms this accuracy, with the simulator achieving a contraction range of 0–79.5%, covering the range of the variability observed across human patients. Figure 8f and supplementary **Movie S3** provide direct visualization of the LAA ostium motion during atrial systole and diastole using endoscopic imaging. The simulator captures the cyclic contraction and relaxation of the ostium, closely mimicking the anatomical behavior observed in humans (e.g., Patient 1), demonstrating the simulator’s capability to recreate intricate LAA geometrical changes during the cardiac cycle.

Figure 8g compares LA area changes during diastole and systole across porcine, human, and simulator data. The echocardiographic images highlight similar LA contraction and relaxation patterns across all three cases, with the simulator showing comparable inward wall motion during systole. Quantitative analysis of the LA area change (percentage reduction from diastole to systole) reveals no significant difference among the porcine (37.4 ± 3.8%), human (39.2 ± 4.1%), and simulator (36.8 ± 4.7%), indicating the model’s ability to replicate atrial function with high fidelity.

The replication of LA hemodynamics was validated by comparing LAP waveforms from porcine in vivo measurements and the simulator (Fig. 8h). Both datasets exhibit similar characteristic waveforms, reflecting the pump, reservoir, and conduit phases of the atrial cycle. The peak LAP values of the simulator closely matched those observed in the porcine model, with the simulator maintaining physiologically relevant pressure ranges.

## Discussion

This study introduces a multimodal framework that integrates experimental and computational approaches to replicate left atrial function with high fidelity in sinus rhythm, atrial fibrillation, and atrial flutter for testing stroke prevention strategies. Combining a soft robotic benchtop simulator, lumped parameter modeling, and finite element analysis, the framework overcomes the critical limitations of traditional methods such as reliance on animal models and cadaveric specimens. This model suite recreates physiological hemodynamics and motion, with the potential to improve LAAO outcomes by (i) facilitating device testing and development in a representative and physiologically relevant model, (ii) enabling patient-specific procedural planning and simulation, and (iii) serving as a tool to better understand left atrial hemodynamics and post-procedural physiological changes following LAAO.

The soft robotic benchtop simulator replicates the anatomy, hemodynamics, and biomechanics of the left atrium and left ventricle of the heart. The use of 3D-printed LA geometries, coupled with soft robotic actuators, allows precise recreation of contractile motion, including the atrial kick, which is often neglected in existing models. Besides 3D printing, the models of the LA and LAA can potentially also be created using simultaneous utilization of additive manufacturing and traditional casting/molding techniques using elastomers (e.g., silicone) or Polyvinyl Alcohol (PVA) cryogel, to accurately replicate tissue mechanical properties and surface finish, ensuring adaptability to user needs. Integration of a soft robotic left ventricle further enhances the system by overcoming the limitations of traditional pulsatile pumps, which exhibit paradoxical behavior when attached to passive ventricular models. The soft robotic left ventricle was validated in a hybrid synthetic-biological configuration with a swine circulatory system. In this setup, the soft robotic LV replaced native ventricular contraction to sustain systemic circulation, integrating with the native vasculature. This validation highlights the robustness of the mock flow loop and its ability to recreate clinically relevant hemodynamics in a dynamic and reproducible manner for benchtop testing. Importantly, the echocardiography and MRI-compatible design of the simulator allows detailed structural imaging and measurement of intra-atrial flow dynamics, further enhancing its utility in studying complex conditions such as AF. Additionally, the use of an internal imaging system with a clear blood-mimicking fluid allows for direct visualization of various internal structures in a way that surpasses current in vivo methods. While intracardiac echocardiography (ICE) is the closest available option for real-time cardiac imaging in patients, direct endoscopic visualization is not feasible outside of open cardiac surgery, where surgical manipulation alters hemodynamics. In contrast, this model provides unobstructed, high-fidelity visualization of intra-cardiac structures and flow dynamics under controlled conditions, making it a superior alternative to in vivo studies for device testing and mechanistic research.

The LPM and FEA components of the framework complement the experimental simulator by providing mechanistic insights into systemic and localized hemodynamic phenomena. The LPM enables predictive modeling of hemodynamic changes, such as atrial inflow, outflow, and pressures pre- and post-LAAO intervention. The FEA model adds granularity by quantifying biomechanical stress distributions and pressure-volume relationships, elucidating the structural and mechanical implications of atrial fibrillation. These computational tools bridge the gap between bench testing and clinical applicability, enabling comprehensive analyses that extend beyond experimental models alone. Although the results from the three models are presented independently in this manuscript, the framework can be integrated into a hybrid experimental-computational configuration, enabling dynamic data exchange, cross-validation, and the use of one model’s outputs as boundary conditions for another. This interconnected approach could offer comprehensive insights, addressing multifaceted hypotheses and bridging system-level and localized analyses in the future.

The suite of benchtop and computational framework successfully simulated the reservoir, conduit, and pump modes of the LA and replicated the clinical structure-function parameters of pressure, volume, and flow. By adjusting circuit and control system parameters and by selectively activating-deactivating soft robotic actuators, we demonstrated the ability to simulate a wide range of atrial dysfunctions. These included clinically relevant abnormalities in contractility, compliance, LAA emptying fraction, arrhythmias, and LAA velocity, with the resulting effects captured in systemic hemodynamic parameters such as left ventricular filling and cardiac outflow. This capability makes the platform uniquely suited for studying not only normal physiological conditions but also pathological scenarios, offering insights into the systemic impacts of left atrial and left atrial appendage dysfunctions.

The ability to replicate device settings, systemic hemodynamics, and localized biomechanical stresses demonstrates the robustness of the entire framework, making it suitable for diverse applications, including intervention testing, clinical research, procedural planning, and education. For instance, this flexible framework involving a suite of three independent models can allow users to adopt specific components to suit their unique needs. Device designers can leverage the benchtop platform to test early prototypes of their devices under realistic conditions. Clinical researchers can employ the LPM and FEA platforms to investigate specific research questions, such as understanding the impact of disease progression or interventions on systemic and localized hemodynamics. Clinicians can use the simulators for procedural planning, refining procedural techniques, surgical trainee practicing, and educating patients by demonstrating intervention dynamics on a beating heart model ^38^.

The ability to simulate patient-specific anatomy and hemodynamics can allow clinicians and trainees to practice device deployment and evaluate potential outcomes before performing actual procedures. This capability is particularly relevant for LAAO, a highly complex intervention requiring precise device placement and a deep understanding of patient-specific anatomy and flow dynamics. Clinical developers can leverage this beating heart simulator to train physicians in rehearsing complex clinical device deployments, ensuring precision and confidence in real-world procedures ^39^. Furthermore, the capability to generate datasets representing both healthy and diseased states paves the way for this framework to be adopted in regulatory settings, potentially accelerating device development in the future ^12^. In line with V&V40 guidelines, such experiments are essential for verifying and validating in silico models, increasing their credibility for use in in silico trials and regulatory decision-making. Achieving this will require interdisciplinary partnerships and standardization, enabling a unique, synergistic opportunity for collaboration among scientists, clinicians, regulatory agencies, and device manufacturers. Such partnerships will not only enhance innovation but also expedite progress toward improved patient outcomes and advanced medical solutions.

This work highlights the utility of the simulator for preclinical testing and mechanistic understanding in atrial fibrillation. However, this workflow can be equally adaptable for other LA-focused interventions ^40,41^. For instance, heart failure with preserved ejection fraction (HFpEF) represents a complex clinical syndrome characterized by symptoms of heart failure, a preserved ejection fraction, and evidence of diastolic dysfunction. Unlike heart failure with reduced ejection fraction (HFrEF), where several evidence-based therapies have shown significant benefits, effective treatments for HFpEF remain limited, emphasizing a critical unmet medical need. One emerging approach involves designing devices to reduce diastolic pressure in the left ventricle by strategically alleviating preload from the left atrium ^41–43^. These devices create a shunt that allows blood to flow directly from the left atrium to the aorta, bypassing the left ventricle. Ensuring the efficacy and safety of such devices necessitates rigorous preclinical testing to optimize their design and functionality. Herein lies another potential role of our benchtop beating left heart simulator, designed to replicate the biomechanics and hemodynamics of the left atrium. This simulator’s ability to mimic patient-specific left atrial anatomy and function, along with the entire left-sided circulation, can enable a thorough evaluation of how shunt devices interact with various anatomical configurations and disease states. By recreating the precise anatomical and physiological conditions of the left atrium, including the hemodynamic challenges of HFpEF, the simulator serves as a tool for assessing device performance under highly controlled yet clinically relevant conditions.

Notably, all intracardiac LAAO devices require transseptal puncture for deployment, a step that is conventionally left unclosed due to its tendency to seal spontaneously. However, in some cases, persistent interatrial shunting occurs, which is usually inconsequential but occasionally problematic. Understanding how transseptal punctures behave in different hemodynamic states could be an area for further study using this framework, particularly in the context of post-procedural atrial remodeling and long-term interatrial shunt persistence.

Beyond simulating LA hemodynamics and motion, the simulator also supports comprehensive hemodynamic modeling of the entire left-sided cardiac circulation. The mechanical valves integrated into the simulator can be replaced with bioprosthetic or novel valve designs, allowing it to replicate pathological conditions such as mitral and aortic valve diseases ^44^. This capability extends to testing valve replacement scenarios. For example, the mechanical mitral valve in the model, which precisely replicates mitral inflow during diastole and occlusion during systole, can be substituted with bioprosthetic valves to investigate various mitral valve disease states and repair or replacement interventions ^45^. This adaptability makes the soft robotic simulator a versatile platform for studying valve interventions and their effects on hemodynamics, offering significant potential to advance device innovation and therapeutic strategies for complex cardiac conditions.

Stress modeling using the computational FEA model could aid in staging AF and refining patient selection for rhythm control. With rising AF ablation efficacy and expanding indications, there is an increasing need to predict who will benefit most. While broad rules exist—e.g., massive biatrial enlargement predicts poor rhythm control success—many patients fall into a clinical gray zone. If stress concentration patterns from computational modeling could help stratify patients for ablation, this could provide a valuable predictive tool. Future work integrating biomechanical stress markers with electroanatomic and clinical data may enhance AF management.

Despite its strengths, the framework has certain limitations that warrant further investigation. First, while the soft robotic simulator effectively replicates atrial wall motion and hemodynamics, its capability to simulate long-term physiological adaptations to chronic conditions remains limited. Future iterations could incorporate feedback-driven adjustments to simulate disease progression and chronic remodeling. Additionally, while the validation studies demonstrated physiological accuracy, further testing across a wider range of patient-specific geometries and pathologies is needed to generalize the findings. The computational models could benefit from integration with machine learning algorithms to enhance predictive capabilities. For example, incorporating patient-specific data into LPM and FEA models could enable real-time simulations for personalized treatment planning and time-dependent remodeling in the future. While 2D phase contrast MRI and echocardiography confirm the capability of model for flow and motion measurement, integrating PIV and 4D flow MRI in future could enhance 3D hemodynamic assessment and vortex analysis. Additionally, incorporating thrombogenesis modeling would enable thrombus formation simulations, improving stroke risk prediction and anticoagulation strategy assessment in future.

This multimodal framework represents a transformative approach to studying LA function and interventions, offering unparalleled fidelity and versatility. A model suite capable of recapitulating biomechanics and measuring pressure and flow can serve as a valuable tool for investigating the impact of LA function or dysfunction on hemodynamic conditions that increase the risk of thrombus formation and stroke. By integrating experimental and computational techniques, it addresses longstanding gaps in cardiovascular research, advancing the fields of biomechanics, device development, and personalized medicine. Its applications in device testing, clinical research, procedural planning, and training highlight its potential to improve patient outcomes and drive innovation in AF management. Future advancements and broader validation will further solidify its role as a cornerstone in cardiovascular research and intervention planning.

## Methods

### Fabrication of patient-specific models and integration with McKibben soft robotic actuators

Retrospective cardiac-gated computed tomography angiography (CTA) images were obtained from one healthy individual and one patient with atrial fibrillation (AF). These images were provided by Hospital de la Santa Creu i Sant Pau (Barcelona, Spain) following approval by the institutional Ethics Committee and after obtaining informed consent from the patients. Imaging was performed using a Somatom Force scanner (Siemens Healthineers, Erlangen, Germany) with a biphasic contrast injection protocol. For the control case, full cardiac phase reconstructions were performed at every 1% of the cardiac cycle, except for the 34%- 48% interval, which was not acquired. In the AF patient, images were acquired at every 5% of the cardiac cycle, covering the entire R-R interval (0–99%). The left atria (LA) from both cases were segmented from the CTA images using semi-automatic tools available in 3D Slicer v4.10.11, representing the 0% of the R-R interval, which corresponds to the onset of ventricular systole. From these segmentations, 3D surface meshes were generated using the flying edges algorithm in 3D Slicer. Post-processing steps were applied to refine the LA surface meshes. MeshLab v2021–07 was used to correct intersecting faces and non-manifold edges. Additionally, Autodesk Meshmixer v3.3.15 was employed to define planar surfaces at the ends of the pulmonary veins and mitral valve to ensure anatomical consistency.

The LA models from healthy subjects were fabricated using SIL30, a soft, flexible silicone material provided by Carbon, Inc., and integrated with soft robotic pneumatic actuators. Similarly, the LA and LV models from AF patients were also printed in SIL30.

Soft robotic actuators were customized for specific cardiac regions. For the LA from a healthy patient, three actuators were designed for the anterior and posterior-inferior walls, and a single actuator was created for the left atrial appendage wall. For the LA model derived from an AF patient, only two actuators were implemented in the anterior and posterior-inferior regions to replicate the diminished atrial contractility characteristic of AF, omitting any actuator for the left atrial appendage. Dimensions were carefully measured to ensure the precise fabrication of these elements, and the actuators were fabricated with dimensions tailored for their specific regions. The LV actuators included three circumferential actuators placed at the basal, middle, and apical sections and three helical actuators positioned at an approximate 60-degree angle from the basal plane, as previously optimized ^21^. Flattened McKibben actuators were employed for the circumferential arrangement, while cylindrical McKibben actuators were used for the helical inner layer. Each soft robotic actuator consisted of three core components: a thermoplastic elastomer (TPE) bladder, thermoplastic polyurethane tubing, and a PET expandable braided mesh. The TPE bladder (Stretchlon 200, sourced from FibreGlast Developments Corp.) was formed by heat-sealing two TPE layers on a 3D-printed mold at 300°F for 4 seconds. A 1/8-inch thermoplastic polyurethane tube (from McMaster-Carr) was inserted into the bladder and sealed using Ure-Bond II adhesive (Smooth-On, Inc.). The braided mesh (1/4-inch PET mesh, TechFlex, Inc.) was coated with Ecoflex 00–30 silicone to prevent kinking, and the actuator assembly was hand-sewn with Kevlar thread to ensure structural integrity. The actuators underwent rigorous quality assurance by cycling them approximately 500 times at a pressure of 20 psi, using a custom-built electro-pneumatic control system ^46–49^. This system incorporated electropneumatic pressure regulators and valves (SMC Pneumatics), which allowed precise control of pressure waveforms, heart rate, and systolic/diastolic ratios through analog inputs. To integrate the actuators with 3D-printed cardiac anatomies, they were first encapsulated within a passive silicone matrix layer approximately 3–5 mm thick. After curing, this structure, referred to as the soft robotic myocardium, was adhered to the LV model using Sil-Poxy adhesive (Smooth-On). In the case of the LA, actuators were directly attached to the printed components using Sil-Poxy, and their ends were secured with Kevlar thread for a conformal fit.

### Design and configuration of the mock circulatory flow loop and hemodynamics acquisition

The 3D-printed cardiac models with integrated soft robotic myocardium were connected to a mock circulatory flow loop, simulating left-sided cardiovascular circulation. In this setup, the soft robotic left atrium and left atrial appendage acted as a reservoir, conduit, and pump during atrial diastole and systole, while the soft robotic left ventricle drove systemic blood flow. Directionality of flow was controlled using mechanical valves (St. Jude Medical) positioned as mitral and aortic valves. The mock circulatory loop incorporated hydraulic and mechanical elements, including in-house acrylic compliance chambers to represent systemic and pulmonary compliance. On-off ball valves (McMaster-Carr, 4796K71) were employed to simulate systemic vascular resistance, while a 1:4 straight-flow rectangular manifold (McMaster, 1023N244) divided flow from the reservoir to the four pulmonary veins. During sinus rhythm simulations, the models were actuated at 60 bpm using a custom-built electro-pneumatic control box. The system synchronized atrial contraction (atrial systole) and ventricular systole with programmed time delays. Adjustments to actuation, resistance, and compliance within the loop enabled the simulation of desired left-sided hemodynamics. A blood mimic fluid consisting of 40% propylene glycol by volume in deionized water, with a dynamic viscosity of 4.3 ± 0.8 mPa s, was circulated through the system. Hemodynamic parameters were measured using pressure sensors (PRESS-S-000, PendoTECH) positioned at key locations—pulmonary veins, LA, LAA, LV, and aorta—via 5 F umbilical vessel catheters (CardinalHealth). The sensors recorded biphasic pressure waveforms, while flow measurements at the pulmonary veins, mitral valve conduit, and aorta were taken using an ultrasonic flow probe (ME 13 PXN, Transonic) connected to a T420 multichannel research console (Transonic Systems Inc.). To simulate hemodynamic conditions associated with atrial dysfunction and pathophysiology, the control system was utilized to adjust the actuation input pressure of the soft robotic myocardium, replicating reduced contractility. Alternatively, the control system was programmed to vary the beating rate (40–150 bpm), mimicking atrial arrhythmias. To visualize valve and wall motion, a 1080P HD endoscopic camera (NIDAGE) recorded videos at 30 frames per second. This comprehensive setup provided detailed insights into the mechanical and hemodynamic performance of the models within the simulated cardiovascular environment. The following equation was utilized for evaluating left atrial appendage emptying.

$$LAA\;ejection\;fraction\;\%=\left(\left(LAA_{\text{maximum}\mathrm{area}}-LAA_{\text{minimum}\mathrm{area}}\right)/LAA_{\text{maximum}\mathrm{area}}\right)\times 100$$

### Echocardiographic imaging for structural and functional analysis

The Philips Epiq CVx cardiovascular ultrasound system, equipped with XL14–3 and X5–1 transducers, was utilized for imaging and analysis. Echocardiographic assessments of wall mechanics and valvular motion were conducted using 2-dimensional B-mode imaging. Flow velocity within the left atrium was measured through pulsed wave (PW) Doppler, while the LAA region was evaluated with color Doppler echocardiography to map flow patterns. For precise visualization of flow dynamics, wall mechanics, and valve motion, the transducer was placed directly on the soft robotic heart for epicardial imaging during benchtop experiments. Wall displacement linked to each actuator’s activity in the left atrium and appendage walls was quantified by capturing repetitive wall motion in the region of interest with M-mode echocardiography. Data analysis and visualization were conducted using Q-Vue 2.2 software (Philips), facilitating detailed interpretation of actuator-induced wall mechanics.

### Phase-contrast 2D flow MRI for intra-atrial velocity measurements

To enable compatibility with the MR environment, all ferromagnetic components in the mock circulatory loop were substituted with non-ferromagnetic plastic parts. The pulmonary vein flow splitter was fabricated using VeroBlue material (Stratasys Objet) via 3D printing. The control system responsible for the cyclic actuation of the robotic heart was positioned outside the magnetic resonance imaging (MRI) room to avoid magnetic interference. Imaging was performed using an MRI scanner (3T MAGNETOM Prisma-Fit, Siemens Healthineers). The 2D flow MRI was acquired with a voxel resolution of 1 × 1 × 1 mm, synchronized with a simulated electrocardiogram (ECG) signal mimicking the robotic heart’s beating pattern (60 bpm). Velocity encoding was performed in three directions, focusing on the through-plane component. Imaging parameters for the 2D flow sequence included a repetition time of 126.4 ms, echo time of 5.37 ms, flip angle of 7°, velocity encoding (VENC) range from 40 cm/s to 60 cm/s, temporal resolution of 28 frames per cycle, and a 2D matrix size of 256 × 256. The MRI phase-contrast 2D flow data inside the LA and LAA was analyzed using GTFlow 4.9.21 software (GyroTools). Potential sources of phase offsets, such as eddy currents, were addressed during data processing. This process involved noise masking, velocity anti-aliasing, and eddy current correction to ensure the accuracy and reliability of the velocity maps.

### Device testing for left atrial appendage occlusion and performance assessment

Commercially available occlusion devices, including the WATCHMAN FLX, sizes 24 mm and 27 mm (Boston Scientific), were employed to evaluate hemodynamic changes pre- and post-LAAO and determine the appropriate device size for the patient-specific anatomy derived from AF cases. Device placement within the benchtop simulator was guided in real-time by the clinical user using either echocardiography (Philips Epiq CVx cardiovascular ultrasound system) or a 1080P HD endoscopic camera recording at 30 frames per second. Post-occlusion efficacy was assessed through color Doppler echocardiography, which mapped the flow to verify proper device sizing and positioning. Structural MRI (3T MAGNETOM Prisma-Fit, Siemens Healthineers) was used to confirm and validate the device’s optimal positioning at the LAA ostium. Although MRI is not a standard clinical modality for assessing LAA occlusion efficacy—typically evaluated using post-operative cardiac-gated CT or echocardiography—it was employed in this experimental research setting due to the lack of cardiac-gated CT equipment. MRI allowed for the observation of potential leakage through the device under pulsatile flow conditions and provided qualitative measurements in a controlled environment. To simulate endothelialization and prevent leakage through the polyethylene terephthalate (PET) mesh of the occlusion device, the WATCHMAN FLX devices were coated with a thin layer of silicone (Ecoflex 35 − 00 fast, Smooth-On).

### Lumped parameter modeling for systemic hemodynamic analysis

A lumped parameter model was developed to simulate cardiovascular hemodynamics pre- and post-LAAO, leveraging Simulink/Matlab (MathWorks, Natick, MA, USA) for system modeling and numerical simulations. The cardiovascular system was represented as a network of resistive, capacitive, and inertial elements, capturing the hydraulic and mechanical properties of the systemic and pulmonary circulations, modified from previously published work in literature ^50–54^. Parameters for atrial and ventricular elastance, valvular properties, and vascular compliance were calibrated based on physiological data to ensure accuracy using the parameter estimation tool Simulink/Matlab. The LA was modeled using dynamic elastance, incorporating its three primary functions: reservoir, conduit, and pump. A time-varying elastance model was used to represent active contraction during sinus rhythm, expressed as: *LA_Ees* × (*LA_Vt* − *LA_V0*) for LA pressure. The atrial reservoir function was captured using the following equation: *LA_A_res* × (exp(*LA_B_res* × (*LA_Vt* − *LA_V0)*) − 1). Parameter values used for LA are *LA_Ees* = 0.45 mmHg/mL (end-systolic elastance), *LA_V0* = 0 mL (volume at zero pressure), *LA_A_res* = 0.5, *LA_B_res* = 0.049 (dimensionless stiffness coefficients). To simulate post-LAAO hemodynamics, several parameter adjustments were made to reflect the physiological changes. The stiffness for the reservoir function of the left atrium was increased by raising *LA_B_res* to 0.07. Reduced reservoir capacity was modeled by reducing *LA_A_res* to 0.35. A slight reduction in atrial compliance was modeled by increasing *LA_Ees* to 0.7 mmHg/mL. Each simulation was run for 30 s and an ode15s solver was used.

### Finite element modeling for left atrial biomechanics and stress analysis

Finite element analysis was employed to study left atrial biomechanics and the impact of atrial fibrillation versus sinus rhythm on pressure-volume relationships. The dynamic cardiac FEA model was developed using the SIMULIA Living Heart Human Model 2023 and Abaqus 2023, Simulia, Dassault Systèmes ^55,56^. Nonlinear explicit dynamic analyses were performed to simulate the time-dependent deformation and mechanics of the LA under different (patho)physiological conditions. To represent blood flow and simulate its interaction with LA wall mechanics, each cardiac compartment and the systemic circulation were modeled as hydrostatic fluid cavities. Surface-based fluid cavities were incorporated to simulate LA inflow and outflow interactions, enabling dynamic coupling of atrial contraction and relaxation with systemic circulation. The passive mechanical behavior of the LA wall was modeled using the anisotropic hyperelastic material formulation proposed by Holzapfel and Ogden for cardiac tissue ^55^. Active LA tissue mechanics were described using a time-varying elastance model that simulated atrial contraction during sinus rhythm (60 bpm) and the absence of coordinated contraction during AF (120–160 bpm). The mechanical model was meshed using 639176 elements and 199317 nodes and the element types included C3D4, CONN3D2, DCOUP3D, MASS, S3R, S4, SFM3D3, SFM3D4R, and T3D2, ensuring appropriate representation of the complex anatomical and functional structures in the model. The LA cavity pressure and volume changes were simulated over three cardiac cycles of 1 second each to achieve steady-state conditions. For sinus rhythm, LA contraction was dynamically modeled, capturing reservoir, conduit, and pump functions. The electrical model was meshed with 114617 nodes, 424706 elements with DC1D2 and DC3D4 elements type. The baseline model was modified to incorporate electrophysiological and structural remodeling characteristics of AF. Atrial arrhythmias associated with atrial fibrillation were introduced into the model through electromechanical coupling. Three irregular cardiac cycles were simulated with sequential cycle periods of 0.375 s, 0.4 s, and 0.5 s, representing the irregularity of atrial activation patterns in AF. Each cycle consisted of a beat phase followed by a recovery phase, and these steps were repeated sequentially for a total of three irregular cardiac cycles to ensure sustained arrhythmogenic behavior. To simulate atrial fibrillation, the electrical properties of the left atrium were modified from those used to represent normal sinus rhythm. For example, anisotropic conductivity values were reduced to reflect AF-associated slowed conduction and heterogeneity: 20.0, 4.5, 4.5 mm^2^/ms for 120 bpm, 16.5, 4.2, 4.2 mm^2^/ms for 150 bpm, and 15.0, 4.0, 4.0 mm^2^/ms for 160 bpm. Electrical material properties, including refractoriness (*γ*), scaling (*c*), restitution time constants (*μ*1, *μ*2), and oscillation threshold (*α*), were adjusted to mimic AF-related rapid recovery, flattened restitution curves, and chaotic dynamics, ensuring an accurate representation of cycle durations and irregular atrial activity across heart rates. The sinoatrial (SA) node amplitude varied irregularly between − 80 mV and 20 mV, with cycle durations of 500 ms (120 bpm), 400 ms (150 bpm), and 375 ms (160 bpm). Atrioventricular (AV) node parameters reflected disrupted conduction, including reduced delay times (e.g., *t*_delay_ = 42.5 ms for 120 bpm, 31.9 ms for 160 bpm) and shorter repolarization periods (*te* = 95 ms for 120 bpm, 71.25 ms for 160 bpm). Rise times (*t*2) ranged from 1.9 to 7.5 ms, and threshold activation potentials (𝐸POT Act = 10 mV ± 1 mV) included random perturbations to simulate chaotic atrial activity. The active and passive material properties of the LA were also modified to reflect the stiffness, reduced relaxation, and increased passive tension characteristic of AF. Mechanical analysis was achieved by mapping electrical outputs to drive myocardial contraction dynamics. Biomechanical Mises stress distributions in the LA wall were analyzed at end-diastole and peak systole, while pressure-volume data were extracted to compare functional differences between sinus rhythm and AF conditions.

### Validation of left atrial model functionality using human imaging data

Retrospective anonymized human echocardiographic imaging data (n = 7) acquired through transesophageal echocardiography (TEE) and tabular hemodynamic data (including LA volume, left atrial mean pressure values, and pressure for v-wave and a-wave; n = 3) were provided via a collaborative partnership with Cedars-Sinai, Los Angeles, USA (IRB approval number STUDY00002705). Left atrial (n = 3) and LAA (n = 7) wall motion and contractility were analyzed using Q-Vue 2.2 software (Philips). All these patients had a history of atrial fibrillation and were considered candidates for left atrial appendage occlusion device implantation. The analysis focused on calculating the percentage area change of the left atrium between systolic and diastolic phases to validate the contractility range achieved by the soft robotic actuators.

### Animal handling, surgical procedures, and in vivo hemodynamics in porcine models

To confirm the physiological capabilities of the model and validate its biomechanics and hemodynamics, experiments were conducted using an acute porcine model (n = 3). LALV The animal experiments adhered to ethical guidelines outlined in the National Research Council’s Guide for the Care and Use of Laboratory Animals and were conducted under MIT Institutional Animal Care and Use Committee protocol #2311000601. Three Yorkshire swine (60–80 kg, sourced from CBSET, Inc.) were used. Animals were intubated, placed on mechanical ventilation, and maintained under general anesthesia (2–3% isoflurane). Arterial and venous femoral lines were inserted for systemic blood pressure monitoring and medication delivery, respectively, and a median sternotomy was performed to access the thoracic cavity. To measure pressures and volumes, a Transonic Scisense ADV 500 Large Animal pressure-volume (PV) System console with a 5F straight-tip PV loop catheter (Model: SCISENSE ADVANTAGE Large Animal PV Foundation System, V.5.0) was inserted through a catheter secured in the left atrium to collect data from the LA and LV respectively as needed. Catheters were secured with purse-string sutures, and flow probes (ME 13 PXN, Transonic connected to a T420 multichannel research console, Transonic Systems Inc.) were positioned around the aorta to measure cardiac outflow. Data acquisition was conducted with a PowerLab 35 series system (ADInstruments) at a sampling frequency of 1 kHz. Real-time monitoring and analysis were performed using LabChart Pro v8.1.16 (ADInstruments), and pressure and flow data were processed with a 10 Hz low-pass digital filter to remove high-frequency noise. Epicardial imaging was performed using 2D echocardiography (Philips Epiq CVx cardiovascular ultrasound system) to validate LA contractility, with visualization and analysis conducted in Q-Vue 2.2 software (Philips).

The validation process began with collecting invasive hemodynamic data, including left atrial pressure, LA volume, left ventricular pressure, systemic blood pressure, and cardiac outflow during native, healthy conditions. To mimic left atrial appendage occlusion and evaluate its hemodynamic effects for validating the lumped parameter model, the pig’s LAA was externally clipped using a hemostat clamp. Pre- and post-clamp hemodynamics were measured using the PV catheter positioned within the left atrium. After collecting native baseline hemodynamic and wall biomechanics data from live pigs, the native left heart was surgically bypassed with the soft robotic LV to assess the model’s capability to replicate systemic hemodynamics in the porcine circulatory system. The bypass procedure involved directing inflow to the soft robotic LV by cannulating the pig’s left atrium and connecting it to the inflow graft of the robotic LV. This graft included a St. Jude mechanical valve functioning as a surrogate mitral valve to regulate flow directionality. The outflow graft, also equipped with a St. Jude mechanical valve to mimic the aortic valve, was connected to the pig’s native aorta via a cannula. The animals were anticoagulated with 5,000 units of heparin IV, and after the bypass circuit was established, the animals were humanely euthanized with pentobarbital at a dose of 100 mg/kg body weight. This step ensured the exclusive evaluation of the soft robotic LV’s performance by eliminating any contributions from the native LV. The soft robotic LV was then actuated right away to assess its ability to generate systemic pressures and flows. This experiment tested whether the soft robotic LV could independently recreate systemic hemodynamics within the swine circulatory system of realistic preload, afterload, vascular resistance, and compliance values.

## Data Availability

The data is available in the main article and supplementary information. All additional data can be requested from the corresponding author.
